# Microparticulated Mefenamic Acid with High Dispersion Stability for Pediatric Dosage Form

**DOI:** 10.3390/children9060861

**Published:** 2022-06-09

**Authors:** Moe Yamazaki, Emi Shimamura, Takehisa Hanawa, Yayoi Kawano

**Affiliations:** Faculty of Pharmaceutical Sciences, Tokyo University of Science, Tokyo 162-8601, Japan; moe.yamazaki.may.22@gmail.com (M.Y.); 3a13482@alumni.tus.ac.jp (E.S.); y.kawano.tus@gmail.com (Y.K.)

**Keywords:** suspension, mefenamic acid, particle size, hydroxypropyl cellulose, dispersibility, wet-milling

## Abstract

Mefenamic acid (MFA), a water-insoluble drug, is used as a suspension in the medical field, but it requires shaking before using to disperse MFA content in the suspension. In previous studies, trials to prepare MFA suspension with high dispersion stability by atomizing MFA by the wet-milling method. However, HPC is used for atomizing MFA. Therefore, the optimum concentration and molecular weight for atomizing MFA have not been investigated. In this study, we investigated the optimum molecular weight and concentration of HPC for the micronization of MFA. As a result, MFA particles became fine particles by adding SDS, and the particle size was also smaller than that of HPC alone. In addition, the suspension with the highest dispersion stability can be obtained when a mixed solution of 1.0% HPC-SL and 0.12% SDS aqueous solution is used. Therefore, this study considers that the addition of SDS and 1.0% HPC-SL aqueous solution are optimal for improving the dispersion stability of the MFA suspension.

## 1. Introduction

Oral administration is the most common method of drug administration because it is the most convenient and usually the safest, and least expensive [1]. Various oral dosage forms are known, including tablets, capsules, sprays, and liquids. Liquid dosage forms are suitable for pediatric patients and the elderly with impaired swallowing ability. Unfortunately, liquid preparations account for only about 3% of all pharmaceutical products used in Japan, not a high percentage [2]. Mefenamic acid (MFA) is a frequently used drug in the pediatric field. MFA, 2-(2,3-dimethyl phenylamine) benzoic acid, is a nonsteroidal anti-inflammatory drug prescribed as an analgesic, anti-inflammatory, and antipyretic [3]. MFA is available in capsule, powder, granule, and syrup formulations, but the syrup formulation is more suitable for children with immature swallowing capabilities.

Furthermore, the solubility of MFA in water is extremely low at 0.80 µg/mL. Therefore, syrups containing MFA are sold with MFA dispersed in a dispersant, but MFA readily precipitates and accumulates at the bottom of the container. Thus, the user must thoroughly shake the MFA when taking the drug to ensure a homogeneous dispersion system, which may reduce patients’ adherence to the medication [4]. These factors have led to the need to develop MFA-containing syrups with high dispersion stability.

Dispersion stability of the dispersed material can be evaluated using the Stokes equation as an indicator of the sedimentation rate of particles with viscosity and particle diameter as parameters. That is, (1) a decrease in particle diameter and (2) an increase in the viscosity of the suspension will decrease the sedimentation rate of the dispersed material. Therefore, the objective of this study was to prepare MFA suspensions with high dispersion stability by (1) reducing the particle size of MFA.

Methods for preparing dispersions with high dispersion stability, such as nanocrystallization by mixing and milling in the presence of appropriate additives, are known to reduce particle size. Among these techniques, wet milling with water-soluble polymer formulations and surfactants is a method with minimal sample loss. In addition, various ways have been reported for convenient preparation at the laboratory level [5].

It is generally known that when API is wet-milled alone, the surface area increases as the particle size decreases, and the destabilized particles re-agglomerate to reduce the interfacial energy. Therefore, in this study, using water-soluble polymers as additives, we attempted to prepare suspensions that (1) prevent particles from approaching each other by surrounding them and (2) maintain a stable dispersion system for a long time by decreasing the sedimentation rate of particles by increasing the viscosity of the dispersion medium.

Since it has been reported that water-soluble polymers adsorb on the surface of nanoparticles and inhibit particle aggregation through steric hindrance [6], it is thought that a larger molecular weight or concentration of water-soluble polymers would increase the layer of polymers adsorbing on the nanoparticles and increase the particle aggregation inhibitory effect. However, although our laboratory has attempted wet milling using hydroxypropyl cellulose (HPC), a water-soluble polymer commonly used as an agglomeration inhibitor for primary drug particles in wet milling [7,8], the water-soluble polymer’s molecular weight and concentration on the suspension’s dispersion stability have not been investigated.

Therefore, in this study, the influence of water-soluble polymers’ molecular weight on suspensions’ dispersion stability was examined using three types of HPC (HPC-SSL, HPC-SL, and HPC-L) with the same structure. Still, different molecular weights and an attempt was made to prepare a suspension with high dispersion stability. In addition to the two-component wet milling of the primary drug and water-soluble polymer, three-component wet milling was performed by adding sodium dodecyl sulfate (SDS), an anionic surfactant commonly used in wet milling, which is present around the primary drug particles and inhibits particle aggregation by creating an electrostatic repulsive effect. The feasibility of further MFA particle size reduction was investigated. Furthermore, by selecting HPC with the optimal molecular weight for milling and varying the amount of HPC added, the concentration of water-soluble polymers on the dispersion stability of the suspension was examined, and an attempt was made to prepare a suspension with higher dispersion stability for a pediatric dosage form

## 2. Materials and Methods

### 2.1. Materials

Mefenamic acid (MFA) was purchased from Sigma-Aldrich Japan (Tokyo, Japan). Hydroxypropyl cellulose (HPC) with three different molecular weights (SSL: 40,000, SL: 100,000, L: 140,000) was purchased from Nippon Soda Co. Sodium dodecyl sulfate (SDS) was purchased from Wako Pure Chemical Industries, Ltd. (Osaka, Japan). Purified water was used as the dispersion medium—all other chemicals and solvents are of analytical reagent grade.

### 2.2. Preparation of Mixed Milled Materials (GM) by Wet Milling

Components of formulations evaluated in this study were listed in Table 1. An amount of 0.6 g of MFA was added to a solution of HPC-SSL, HPC-SL, and HPC-L prepared at various concentrations and SDS, 60 g of zirconia beads was added, and the mixture was stirred at 500 rpm for 24 h using a propeller-type stirrer (Three-One Motor, Shinto Scientific Co., Ltd., Tokyo, Japan). After mixing, the zirconia beads and suspension were sieved through a 166-mesh sieve (90 μm aperture, Tokyo Screen Co., Ltd., Tokyo, Japan) and washed with purified water. The dispersant concentration in the suspension after sieving was 0.5 *w*/*v*%.

### 2.3. Evaluation of Physicochemical Properties of MFA Suspensions

The physicochemical properties of each MFA suspension were evaluated immediately after preparation and after one and two weeks of storage at 4 °C. In addition to the MFA suspension, we also assessed the physical properties of the MFA bulk powder, the PM of each sample, and the MFA suspension (Pontal Syrup^®^) that was already commercially available.

### 2.4. Measurement of Particle Size

For particle size, 50 mL of the sample was suspended in 10 mL of ultrapure water. The average particle size was measured at room temperature (25 °C) using a particle size measurement system (ELSZ-2/ELSZ-2000-m2000ZS, Otsuka Electronics Co., Osaka, Japan) cumulant analysis.

### 2.5. Observation by Scanning Electron Microscopy (SEM)

An amount of 100 μL of sample suspension was added to 900 μL of purified water and centrifuged at 15,000 rpm (21,652.65× *g*) for 5 min using an ultracentrifuge (HIMAC CP80MX, rotor: Angle Rotor R80AT, (Koki Holdings, Inc., Tokyo, Japan). After removing the supernatant, 1 mL of purified water was added, and the suspension was sonicated for 2 min and centrifuged again at 15,000 rpm (21,652.65× *g*) for 5 min. After removing the supernatant, 1 mL of purified water was added, suspended by sonication for 2 min, and dried. Dried samples were sputtered with platinum on a sample table and measured at an acceleration voltage of 20 kV. Scanning electron microscopy (SEM) was performed using a JSM-6390LA (JEOL Ltd., Tokyo, Japan).

### 2.6. Differential Scanning Calorimetry (DSC)

Differential scanning calorimetry (DSC) was performed on the MFA, HPC, the physical mixture of samples F’1, F’2, and F’3, and the MFA suspension of models F1, F2, and F3 using DSC-60 Plus (Shimadzu Co., Ltd., Tokyo, Japan). Each piece was measured at a temperature increase rate of 3 °C/min, in the range of 0 to 240 °C. Alumina was used as a reference.

### 2.7. Powder X-ray Diffraction Measurement (PXRD)

Powder X-ray diffraction measurements were performed using a powder X-ray diffractometer (RINT2000, Rigaku Corporation, Tokyo, Japan) with a CuKα_1_ radiation source, Cu target, Ni filter, 40 mV voltage, 40 mV current, 2°/min scanning speed, and 4 times integration.

### 2.8. Fourier Transform Infrared Spectrophotometry (FT-IR)

Samples were centrifuged at 30,000 rpm (86,610.6× *g*) for 30 min at 30 °C in an ultracentrifuge to separate the aqueous and solid phases. A total reflection Fourier transform infrared spectrophotometer (U-ATR, Perkin Elmer Japan Inc., Kanagawa, Japan) was used. The sample thickness was 1.0 mm. The scanning range was 500–4000 cm^−1^.

### 2.9. Dispersibility Evaluation by Turbiscan

Dispersion stability evaluation was performed using the solution stability evaluation device Turbiscan MA2000 (Formulaction, Toulouse, France). At room temperature (about 25 °C), a pulsed near-infrared LED with a wavelength of 880 nm was irradiated while moving from the bottom to the top of the sample tube, and the intensity changes in transmitted light (T) and backscattered light (BS) were measured. The measurement interval was every 24 h for 14 days.

## 3. Results and Discussion

### 3.1. Particle Size Distribution of Various Samples

**Figure 1:**
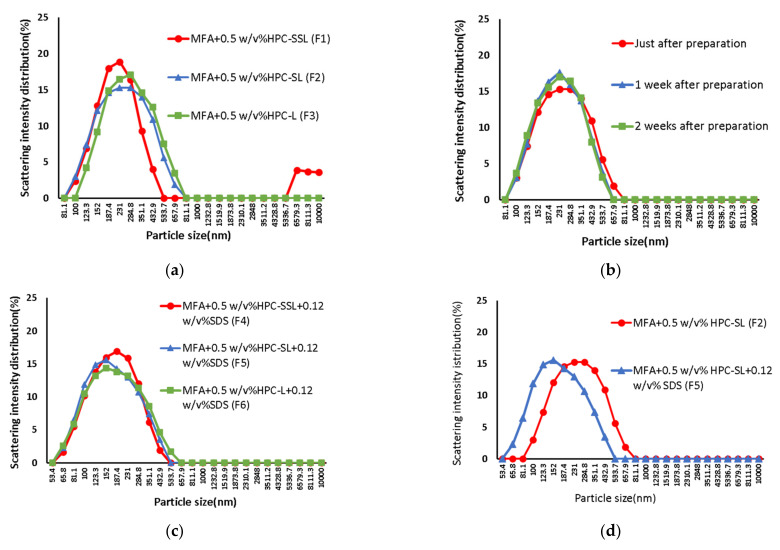

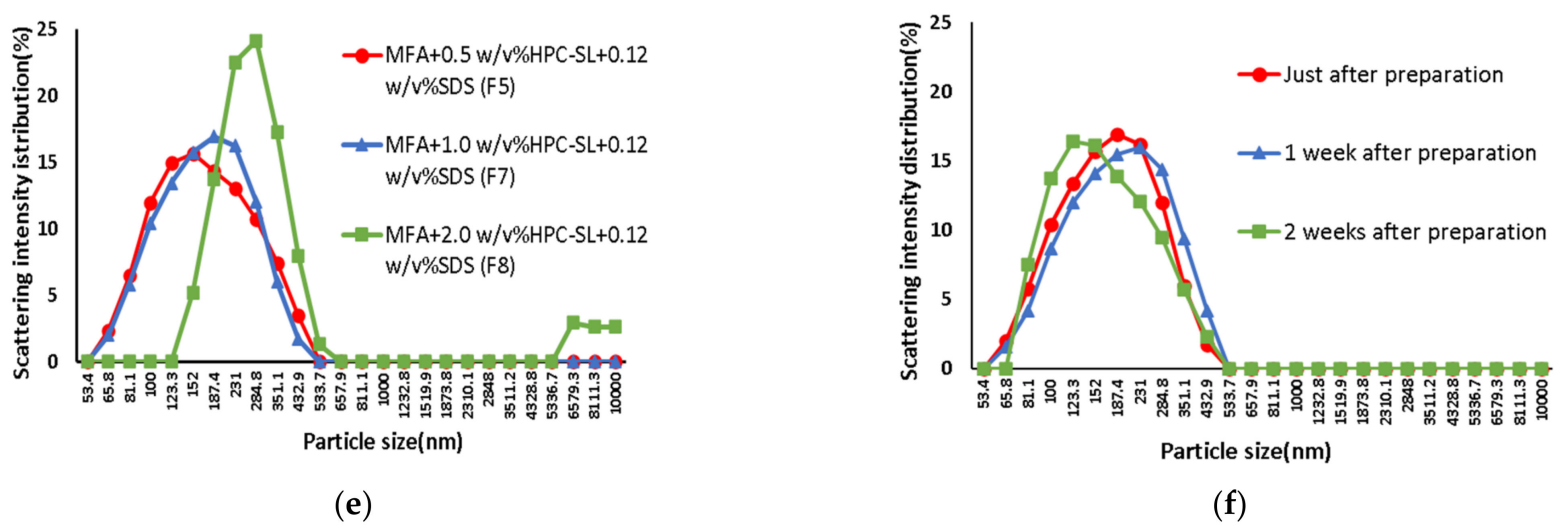
Size distribution of MFA particles in each sample: (**a**) F1-F3 immediately after preparation, (**b**) F2 immediately after preparation to 2 weeks after preparation, (**c**) F4–F6 immediately after preparation, (**d**) F2 and F5 immediately after preparation, (**e**) F5, F7, and F8 immediately after preparation, (**f**) F7 immediately after preparation to 2 weeks after preparation.

The particle size distribution of each milled sample is shown in Figure 1. The particle size distribution of the samples wet-milled with two components, MFA and HPC, in suspension immediately after preparation was bimodal for HPC-SSL (F1) and unimodal for the others, so the size distribution of MFA particles in suspension with HPC-SL and HPC-L was unimodal, and high dispersion stability can be expected (Figure 1a).

Next, a comparison of the two samples (F2 and F3) that showed unimodal distribution showed that the particle size of MFA immediately after preparation with HPC-SL was 219 nm, the smallest particle size; it seems that the viscosity of aqueous HPC solutions increases with increasing molecular weight and that the same concentration of HPC-SL aqueous solution is less viscous than HPC-L aqueous solution, the collision rate between MFA and ZB during milling This was thought to be due to an increase in the collision velocity between MFA and ZB during grinding. The particle size measurement of the HPC-SL suspension, which showed the smallest particle size among the three species, showed no change in particle size over time, suggesting that the aggregation of MFA particles over time was suppressed (Figure 1b).

Furthermore, when milling with three components with SDS, the smallest particle size was obtained in the sample with HPC-SL, which was smaller than that obtained when milling with two components (Figure 1c,d). This may be because the addition of SDS improved the affinity of MFA to the HPC solution, enabling more efficient milling of MFA. In our previous study [9], when cefditoren pivoxil (CDTR-PI) was wet-milled at various HPCs and concentrations, the smallest particles were obtained at 0.5%, so the concentration was also set to 0.5% in this experimental system. The oral LD50 of SDS is 1200 mg/kg, and we have confirmed that the concentration of SDS used in this experimental system is a safe amount.

When the amount of HPC-SL added was increased, a smaller particle size was obtained using twice the conventional amount of 1.0 *w*/*v*% HPC-SL aqueous solution. As a result, no particle growth was observed (Figure 1f). In contrast, when using four times the amount of 2.0 *w*/*v*% HPC-SL aqueous solution, a bimodal particle size distribution was observed and the particle size (Figure 1e). This may be because the increase in the amount of HPC-SL added also increased the viscosity of the aqueous solution, which reduced the collision velocity between the MFA and ZB, resulting in insufficient progression of the MFA pulverization. The particle size of MFA in various formulations is listed in Table 2.

### 3.2. Morphological Observation by SEM

**Figure 2:**
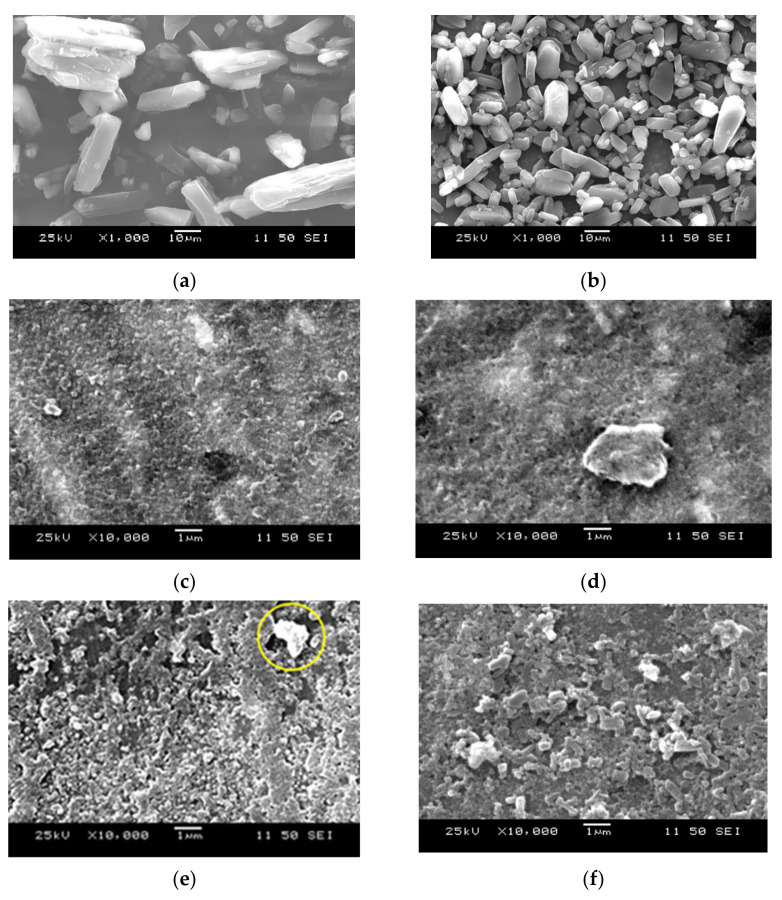

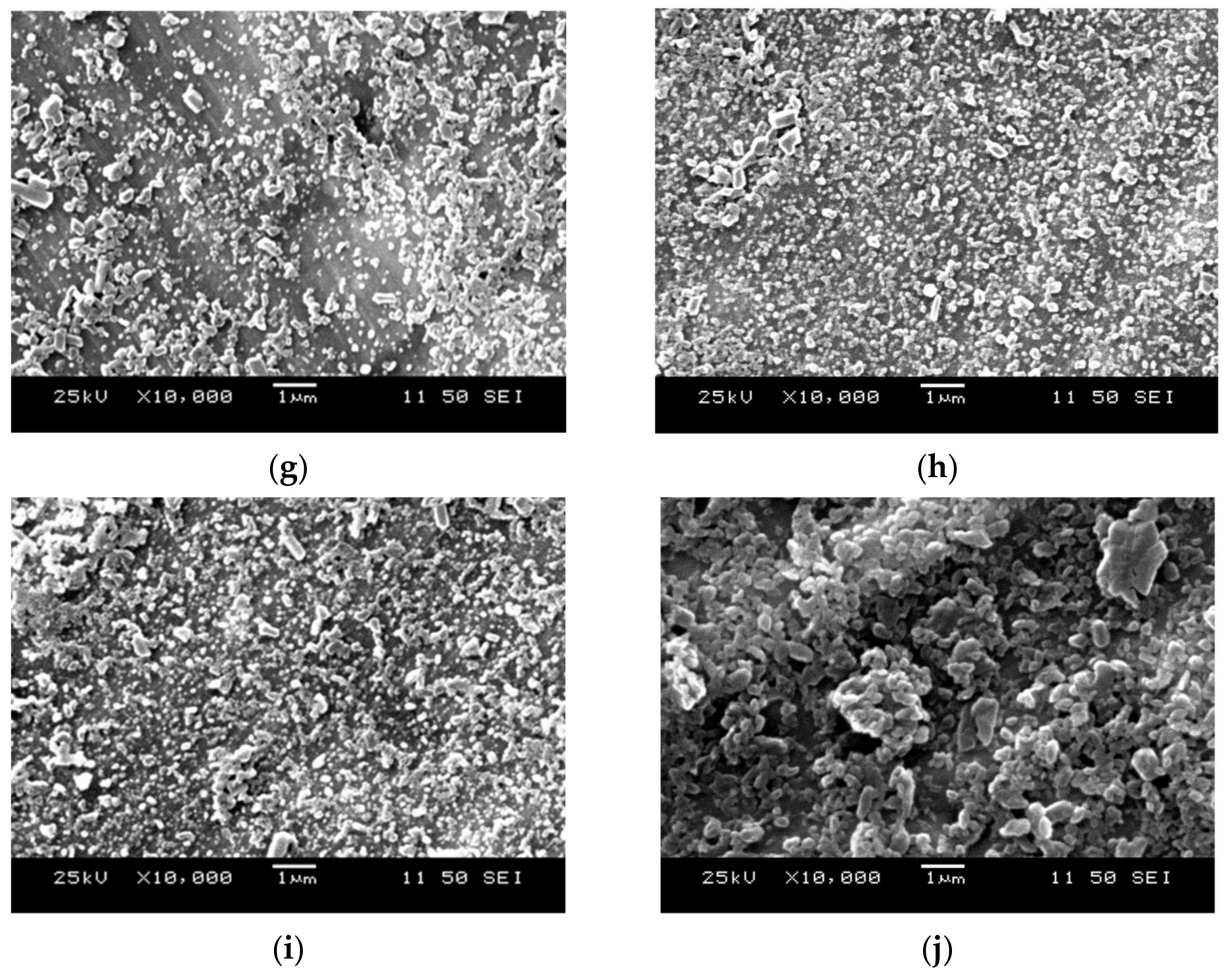
Morphology of MFA particles in each sample immediately after preparation: (**a**) MFA crystals, (**b**) Pontal Syrup^®^, (**c**) MFA+0.5 *w*/*v*% HPC-SSL suspension (F1), (**d**) MFA+0.5 *w*/*v*% HPC-SL suspension (F2), (**e**) MFA+0.5 *w*/*v*% HPC-L suspension (F3), (**f**) MFA+0.5 *w*/*v*% HPC-SSL+0.12 *w*/*v*% SDS suspension (F4), (**g**) MFA+0.5 *w*/*v*% HPC-SL+0.12 *w*/*v*% SDS suspension F5, (**h**) MFA+0.5 *w*/*v*% HPC-L+0.12 *w*/*v*% SDS suspension (F6), (**i**) MFA+1.0 *w*/*v*% HPC-SL+0.12 *w*/*v*% SDS suspension (F7), (**j**) MFA+2.0 *w*/*v*% HPC-SL+0.12 *w*/*v*% SDS suspension (F8).

The results of morphological observation by SEM are shown in Figure 2. The presence of MFA microparticles, which was not observed in the particle size measurement, was observed in the two-component suspension with HPC-SL and HPC-L (Figure 2c–e). This was considered due to the insufficient amount of HPC adsorbed on the surface concerning the microparticulate MFA particles, resulting in aggregation of the MFA particles. In addition, the suspension with two-component milling with HPC showed a non-uniform morphology that made it difficult to recognize the shape of the particles compared to the suspension with three-component milling, suggesting that the addition of SDS could vary more uniformly the microparticulate MFA particles.

On the other hand, in (f), (g), and (h), where SDS was added, agglomerated MFA particles were observed in (f). Still, only microparticulate MFA particles were observed in (g) and (h), and no MFA microparticles were observed. This may be because the addition of SDS increased the affinity between the MFA particles and HPC, and more HPC was adsorbed on the surface of the MFA particles than in the case of milling with HPC alone. Furthermore, in (i) and (j), where the amount of HPC-SL added was increased, the surface of the MFA particles exhibited smoother properties than in (g).

### 3.3. The Thermal Behavior of the Various Samples

**Figure 3:**
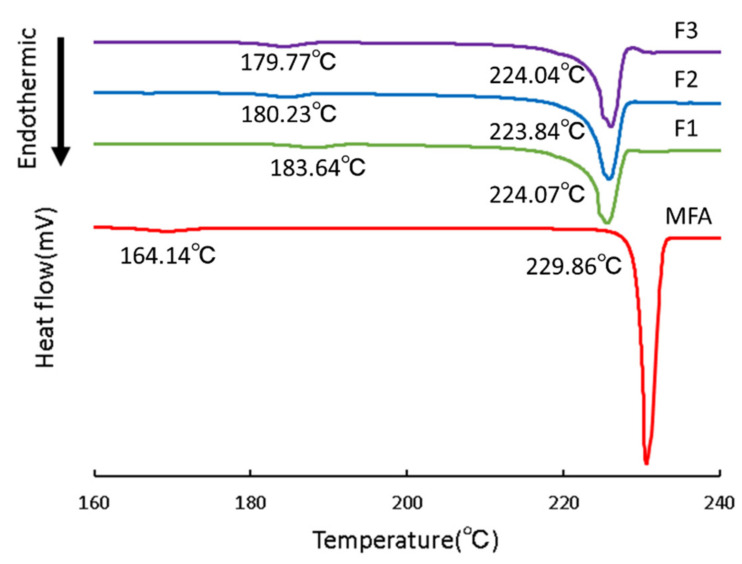
DSC curves of MFA crystals, F1, F2, and F3.

The thermal behavior of the various samples, MFA crystals, F1, F2, and F3, are shown in Figure 3. F4 through F8 are not shown or discussed here because they could not be observed as distinct thermal behaviors due to the coexistence of SDS.

Two endothermic peaks were observed in the DSC curve of MFA crystals, one near 164 °C and the other near 229 °C. MFA is known to have crystal polymorphism [9], and it has been reported that the endothermic peak near 171 °C is the temperature at which Form I crystals transition to Form II crystals, and the peak near 231 °C is the endothermic peak due to thermal decomposition of Form II crystals [10]. In this study, peaks were observed near 164 °C and 229 °C, suggesting that the MFA used in this study is a Form I crystal (Figure 3).

DSC measurements of MFA particles in the MFA suspension obtained by wet milling this MFA crystal with HPC in two components were performed, and the results of the measurements showed that they all maintained the Form I crystal (Figure 3). The temperature of transition from Form I crystals to Form II crystals ranged from about 180 to 183 °C, which was about 15 °C higher after pulverization than that of the original powder. Furthermore, the temperature of MFA transitioning to Form II crystals was about 224 °C, which was 5 °C lower than that of the original powder. These results suggest that the wet-milling process affected the adsorption of HPC around the MFA, making the Form I crystals less sensitive to heat and delaying the transition to Form II crystals.

### 3.4. Powder X-ray Diffraction (PXRD) Measurement

**Figure 4:**
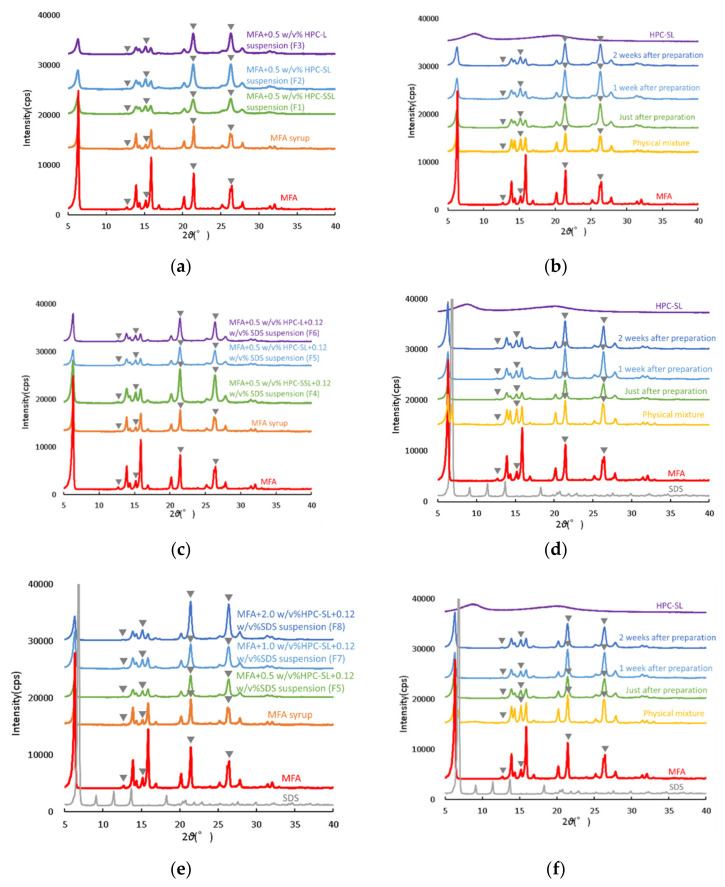
PXRD patterns of various samples: (**a**) F1–F3 just after the preparation; (**b**) MFA particles over time of F2; (**c**) MFA particles just after the preparation of F4–F6; (**d**) MFA particles just after the preparation of F5; (**e**) MFA particles just after the preparation of F5, F7, and F8; (**f**) MFA particle peaks over time in F7.

It is known that MFA crystals have two polymorphs, Form 1 and 2 [11]. The PXRD patterns of various samples are shown in Figure 4. The PXRD patterns of the MFA crystals used in this study showed diffraction peaks at 2θ = 12.5°, 15.5°, 21.5°, and 27.5° (▼ in Figures). The DSC measurements also suggested that the MFA used in this study was a Type I crystal, and these results indicate that the MFA used in this study is a Type I crystal.

The peak intensity of MFA particles (F1–F3) in each sample immediately after preparation when wet milling was performed with two components, MFA and HPC, was lower than that of MFA crystals in all suspensions (Figure 4a).

When focusing on the change over time of the PXRD pattern of the dispersant in the suspension (F2) using HPC-SL, no change in peak intensity, the disappearance of peaks, or appearance of new peaks with time was observed, suggesting that the MFA in the dispersant was in a crystalline state as immediately after preparation. (Figure 4b). Furthermore, when SDS was added (F4–F6), a similar decrease in peak intensity was observed, and the largest decrease in peak intensity was observed in the system using HPC-SL. However, when this sample was stored in a desiccator at 25 °C, an increase in peak intensity was observed over time, suggesting that the addition of SDS may have caused a decrease in peak intensity. The crystallinity of MFA after grinding was decreased by increasing the amount of HPC-SL added by 2. The crystallinity over time also maintained a low peak intensity, suggesting that the growth of MFA crystals was suppressed. Growth was considered to be suppressed. However, the crystallinity of the suspension with 2.0 *w*/*v*% HPC-SL aqueous solution, in which the amount of HPC-SL added was increased fourfold, was higher than that of the other suspensions. This may reflect the crystallinity of larger diameter MFA particles produced by insufficient milling due to the increased viscosity of the HPC-SL aqueous solution.

### 3.5. The Molecular State of MFA in Various Samples

**Figure 5:**
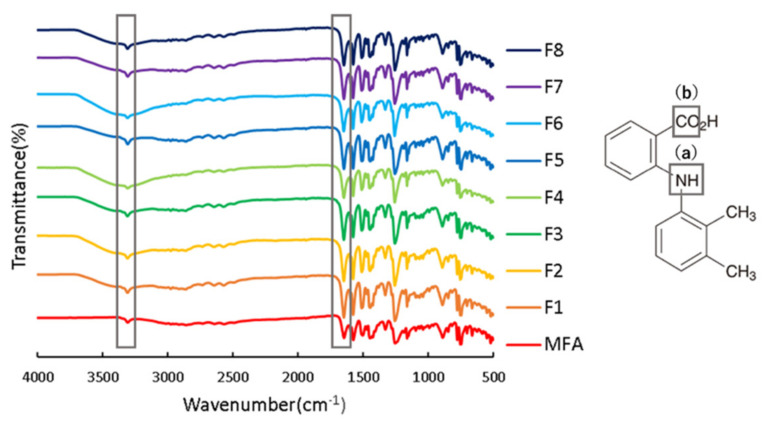
FT-IR spectra of various samples.

The molecular state of MFA in each sample was examined by FTIR (Figure 5).

No change was observed in the position of the amine group stretching vibration-derived peak of MFA at 3306 cm*^−^*^1^ and the carbonyl group stretching vibration-derived peak at 1646 cm*^−^*^1^ before and after pulverization (Figure 5). These results suggest no intermolecular interaction between MFA, HPC, and SDS.

### 3.6. Dispersion Stability Study

**Figure 6:**
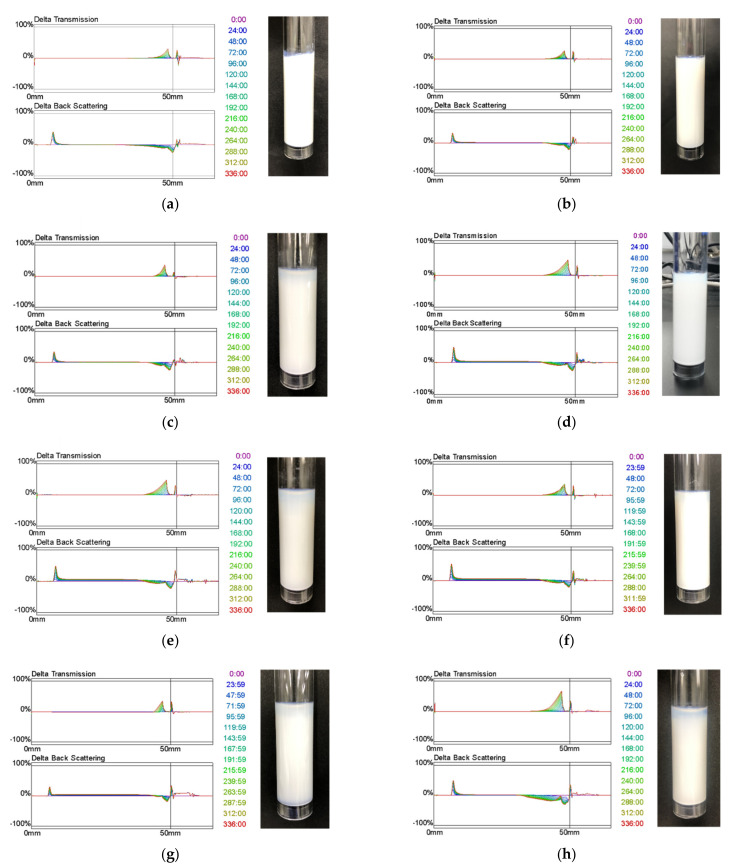
Transmission and backscattering patterns of various suspensions. (**a**) F1, (**b**) F2, (**c**) F3, (**d**) F4, (**e**) F5, (**f**) F6, (**g**) F7, and (**h**) F8.

Dispersion stability is an essential factor in formulation studies of suspensions. The dispersant is dispersed without dissolving in the dispersant [12]. The dispersibility of sample solutions was evaluated over time by transmitted light (T%) and backscattered light (BS%) using a Turbiscan MA 2000. The Turbiscan is a measuring instrument that evaluates the dispersion stability by shining light on the sample and measuring the transmitted and backscattered light. Here, the upper graph (Delta Transmission) in the results shows the deviation change in transmitted light intensity concerning the start of the measurement (t = 0) and evaluates the transmittance of the supernatant as the MFA particles in the suspension settle. The lower graph (Delta Back Scattering) shows the deviation change in the intensity of backscattered light concerning the start of the measurement (t = 0). It evaluates the increase in the amount of backscattered light with the sedimentation of MFA particles in suspension. Therefore, the higher the dispersion stability, the less the changes in transmitted light and backscattered light, and the smaller the peaks in the graph (Figure 6). In light of this, the suspension with HPC-SL showed the lowest Delta Transmission and Delta Back Scattering among the two-component mills, suggesting that it was considered a suspension with high dispersion stability (Table 3).

Second, for the suspensions with SDS, the suspension with 1.0 *w*/*v*% HPC-SL showed the lowest Delta Transmission and Delta Back Scattering values. Conversely, the suspension with 2.0 *w*/*v*% HPC-SL showed the highest value for Delta Transmission value was the largest. This could be because F7 had the smallest particle size and the lowest sedimentation rate from Stokes’ equation.

Furthermore, one way to evaluate dispersion stability using a Turbiscan is to determine the PSI value [13]. The formula is described below.

PSI = (Hs/Hc) × (Bs%)(1)

where Hc is the height of the sample in the sample tube (mm), Hs is the height of sedimentation in the sample tube (mm), and Bs represents the averaged backscattered light (%) at each time (every 24 h).

The smaller the PSI, the higher the dispersion stability of the suspension (Table 3). The PSI results show that the sample with the smallest value is F7, suggesting that F7 has the highest dispersion stability from the numerical aspect and the backscattered light value, and the presence of precipitation by visual observation.

## 4. Conclusions

Syrup dosage forms are easy for children and the elderly to take. Still, in the case of suspension dosage forms, the dispersant must be dispersed uniformly and stably in the dispersing medium for an extended period. In this study, MFA was used as a model API, and wet milling with HPC of various molecular weights was attempted to produce fine particles of MFA particles. In addition, SDS was used as a surfactant, and the significance of the addition of surfactant in wet milling was examined.

As a result, it was found that the system using HPC-SL showed the most stable dispersion stability for an extended period. We believe that wet milling is essential for preparing stable pediatric syrups for the pediatric dosage form.

It is expected that the solubility will be improved by microparticulation, and the bioavailability will be improved as a result. We are currently examining the possibility of improved absorption due to increased solubility and, thus, reduced single doses in another experimental system, and hope to show this along with the results of that study.

## Figures and Tables

**Table 1:** Components of Formulations Evaluated in This Study.

Formulation	HPC Concentration (Grade) (*w*/*v*%)	SDS Concentration (*w*/*v*%)
F1	0.5 (SSL)	0
F2	0.5 (SL)	0
F3	0.5 (L)	0
F4	0.5 (SSL)	0.12
F5	0.5 (SL)	0.12
F6	0.5 (L)	0.12
F7	1.0 (SL)	0.12
F8	2.0 (SL)	0.12

**Table 2:** The particle size of MFA in various formulations.

Formulation	Particle Size (nm)	PDI
F1	152.3 ± 22.1	0.173 ± 0.017
F2	265.7 ± 14.8	0.189 ± 0.006
F3	149.4 ± 1.70	0.169 ± 0.005
F4	160.8 ± 20.4	0.160 ± 0.007
F5	151.3 ± 11.0	0.172 ± 0.032
F6	168.0 ± 14.8	0.176 ± 0.011
F7	270.1 ± 0.49	0.147 ± 0.020
F8	335.1 ± 2.90	0.179 ± 0.028

Data are expressed as mean ± S.D. (*n* = 3).

**Table 3:** Delta transmission, delta backscattering, and PSI of various samples.

Formulation	ΔT (%)	ΔBS (%)	PSI (%)
F1	30.74	41.12	2.43
F2	27.55	33.14	1.58
F3	36.33	33.94	2.07
F4	47.99	47.99	1.67
F5	46.71	49.11	2.26
F6	35.53	54.70	2.72
F7	35.53	29.95	0.80
F8	67.47	49.11	2.34

ΔT; delta transmission, ΔBS; delta backscattering.

## Data Availability

Not applicable.
